# Distal Electroacupuncture at the LI4 Acupoint Reduces CFA-Induced Inflammatory Pain via the Brain TRPV1 Signaling Pathway

**DOI:** 10.3390/ijms20184471

**Published:** 2019-09-10

**Authors:** Chia-Ming Yen, Tong-Chien Wu, Ching-Liang Hsieh, Yu-Wei Huang, Yi-Wen Lin

**Affiliations:** 1 College of Chinese Medicine, Graduate Institute of Acupuncture Science, China Medical University, Taichung 40402, Taiwan; 2 Department of Anesthesiology, Taichung Tzu Chi Hospital, Buddhist Tzu Chi Medical Foundation, Taichung 42743, Taiwan; 3 Emergency and Critical Care Center, E-Da Hospital, Kaohsiung 80708, Taiwan; 4 School of Nursing, College of Nursing, Fooyin University, Kaohsiung 824, Taiwan; 5 School of Medicine, College of Medicine, I-Shou University, Kaohsiung 824, Taiwan

**Keywords:** distal electroacupuncture, inflammatory pain, analgesia, TRPV1, hypothalamus, prefrontal cortex

## Abstract

There is accumulating evidence supporting electroacupuncture’s (EA) therapeutic effects. In mice, local EA reliably attenuates inflammatory pain and increases the transient receptor potential cation channel, subfamily V, member 1 (TRPV1). However, the effect of distal acupoint EA on pain control has rarely been studied. We used a mouse model to investigate the analgesic effect of distal EA by measuring TRPV1 expression in the brain. Complete Freund’s adjuvant (CFA) was injected into mice’s hind paws to induce inflammatory pain. The EA-treated group received EA at the LI4 acupoint on the bilateral forefeet on the second and the third days, whereas the control group underwent sham manipulation. Mechanical and thermal pain behavior tests showed that the EA-treated group experienced inflammatory pain alleviation immediately after EA, which did not occur in the sham group. Additionally, following CFA injection, the expression of TRPV1-associated molecules such as phosphorylated protein kinase A (pPKA), extracelluar signal-regulated kinase (pERK), and cAMP-response-element-binding protein (pCREB) increased in the prefrontal cortex (PFC) and the hypothalamus but decreased in the periaqueductal gray (PAG) area. These changes were significantly attenuated by EA but not sham EA. Our results show an analgesic effect of distal EA, which is based on the traditional Chinese medicine theory. The mechanism underlying this analgesic effect involves TRPV1 in the PFC, the hypothalamus, and the PAG. These novel findings are relevant for the evaluation and the treatment of clinical inflammatory pain syndrome.

## 1. Introduction

The transient receptor potential cation channel—subfamily V, member 1 (TRPV1)—was recently identified as a heat-activated ion channel in the pain pathway [1]. TRPV1 is widely distributed in the peripheral and the central nervous systems, including the non-neuronal mast cells [2]. TRPV1 at peripheral sites is highly expressed in small-diameter C-fibers, which are reported to play a role in pain transduction [3]. TRPV1 can be activated by capsaicin, acidic pH, temperatures above 43 °C, inflammation, and endogenous mediators [4]. It is localized in highly calcium-permeable neuronal membranes, and its activation induces calcium influx to depolarize the membrane and relieve substance P (SP), thereby initiating vessel dilation and permeability, i.e., activating mast cells to release histamine to initiate inflammation [5]. In mice, TRPV1 is increased in the dorsal root ganglion (DRG) and the spinal cord (SC) for up to 28 days after inflammatory pain [6,7], and thermal pain cannot be initiated in TRPV1 knockout mice [8,9]. In addition, CFA- or capsaicin-induced mechanical and thermal pains are prevented in TRPV1 knockout mice [10,11]. Several studies investigated the regulatory role of TRPV1 channels in pain relief. For example, selective TRPV1 blockage significantly reduced thermal pain sensation while increasing body temperature [12,13]. A recent study suggested that TRPV1 increased in the periaqueductal gray (PAG) and the prefrontal cortex (PFC) following an inflammatory challenge [14].

Clinical inflammatory conditions are often mimicked using chemical mediators that initiate local inflammatory responses. Several reagents, such as complete Freund’s adjuvant (CFA), can be injected to induce inflammatory pain. CFA contains inactivated *Mycobacterium tuberculosis*, which induces local inflammation, potentiating cell-mediated immune response [15,16]. CFA injection into the mice’s hind paws induces intense and persistent inflammation at the injection site, which spreads distally [17]. CFA injection has been shown to induce significant mechanical and thermal hyperalgesia, which changes spinal plasticity, including changes in protein content [18]. Local inflammation can activate mechanical and thermal receptors such as TRPV1 [6], acid-sensing ion channels [19], and TRPV4 [20]. Such potentiation can be reversed or prevented by electroacupuncture (EA) [20].

Although acupuncture has been used in Asia for analgesia, its underlying mechanism remains unclear. Several theories have been suggested, such as the associated release of opiates, which desensitize the painful sensitization and reduce proinflammatory cytokines such as tumor necrosis factor-α, interleukin (IL)-1β, and IL-6 [21,22]. Acupuncture may also attenuate the phosphorylated n-methyl-d-aspartate receptor (NMDAR) to relieve pain [23], and it is reported to increase serotonin and norepinephrine, thus reducing spinal NMDAR phosphorylation [24]. Recent studies have indicated that EA can increase the concentrations of endogenous opiates [6], serotonin [25], and adenosine [6], thereby reducing pain. 

The present study aimed to identify the effects of distal EA on inflammatory pain, its underlying mechanism, and its relationship with TRPV1 and associated molecules in the brain. We hypothesized that TRPV1 and related molecules are involved in inflammatory pain and that distal EA reduces inflammatory pain via the regulation of TRPV1 and related molecules in the brain.

## 2. Results

**Figure 1:**
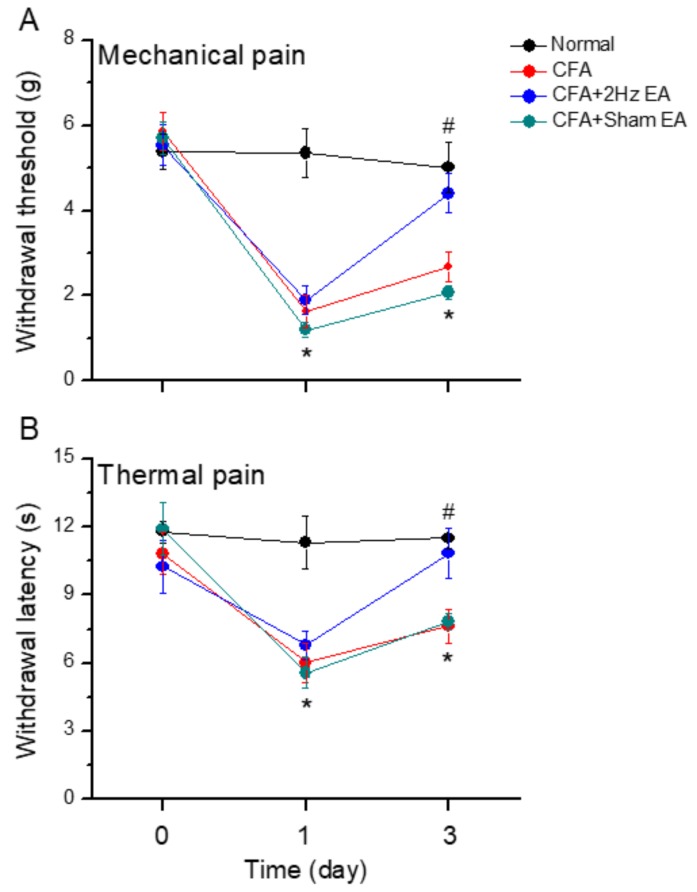
(**A** and **B**). Mechanical and thermal pain thresholds in four groups of mice. Normal saline injection (normal group, *n* =8), complete Freund’s adjuvant (CFA) (CFA-induced inflammatory pain), CFA + 2 Hz electroacupuncture (EA) (CFA-induced inflammatory pain treated with 2Hz EA), CFA + sham EA (CFA-induced inflammatory pain treated with sham EA). *p < 0.05 vs. normal group. #*p* < 0.05 vs. CFA group.

We investigated the efficacy of distal EA to reduce inflammatory pain by comparing responses to the von Frey filament test at baseline and on days 0–3. The control group presented normal values because no CFA-induced mechanical hyperalgesia was performed (Figure 1A, *n* = 8, *p* > 0.05). No significant differences were found among groups regarding baseline measurements. In contrast, the CFA group presented mechanical hyperalgesia (Figure 1A). Similarly, observations of the CFA + 2 Hz EA and the CFA + sham EA groups showed that the level of inflammation-induced mechanical hyperalgesia significantly decreased on Day (D) 1 (Figure 1A, *p* < 0.05, compared with D0, Figure 1A). This effect was reversed on Day 3 in the CFA + EA group, which received low-frequency EA at the LI4 acupoint (Figure 1A, *n* = 8; *p* < 0.05, compared with D1). No significant effects were observed in the CFA + sham EA group (Figure 1A, *n* = 8; *p* < 0.05, compared with D0) compared with the 2 Hz EA group. Similar results were found for the Hargreaves’ test, which assessed thermal hyperalgesia (Figure 1B). At the baseline condition, no significant differences among groups were found regarding the thermal test results (Figure 1B, *n* = 8; *p* > 0.05). Thermal hyperalgesia was not induced in normal animals (Figure 1B, *n* = 6; *p* > 0.05) but was significantly observed in the other three groups, namely, the CFA, the CFA + 2 Hz EA, and the CFA + sham EA groups (Figure 1B, *p* < 0.05, compared with D0). In addition, thermal hyperalgesia was attenuated in the CFA + 2 Hz EA group on Day 3, whereas no attenuation was observed in the sham EA group (Figure 1B).

**Figure 2:**
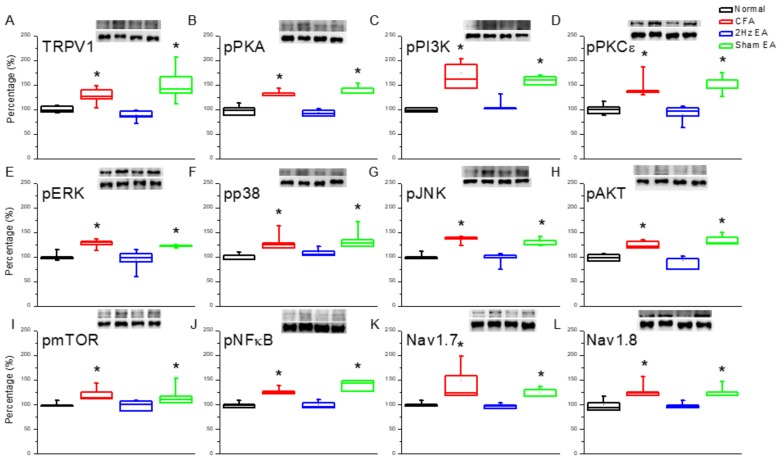
Expression levels of transient receptor potential cation channel, subfamily V, member 1 (TRPV1)-associated signaling pathways in the mice medial prefrontal cortex (mPFC). (**A**) TRPV1, (**B**) pPKA, (**C**) pPI3K, (**D**) pPKCε, (**E**) pERK, (**F**) pp38, (**G**) phosphorylated c-Jun N-terminal kinase (pJNK), (**H**) phosphorylated protein kinase B (pAkt), (**I**) phosphorylated mammalian target of rapamycin (pmTOR), (**J**) phosphorylated nuclear factor κB (pNFκB), (**K**) voltage-gated sodium channel 1.7 (Nav1.7), and (**L**) voltage-gated sodium channel 1.8 (Nav1.8) expression levels in normal, CFA, CFA + 2 Hz EA, and CFA + sham EA (from left to right). Normal = normal mice; CFA = CFA-induced inflammatory pain; 2 Hz EA = CFA + 2 Hz EA. Sham EA = CFA + sham EA. *p < 0.05 compared with the normal group. The western blot bands at the top show the target protein. The lower bands are internal controls (β-actin or α-tubulin).

Next, we investigated the effect of inflammatory pain on TRPV1 and the effect of EA on the TRPV1 receptor and related molecules in the medial prefrontal cortex (mPFC). TRPV1 increased in the PFC of mice with inflammatory pain (Figure 2A, *p* < 0.05, compared with the control group *n* = 6) compared with control mice (Figure 2A, *n* = 6), and this overexpression was significantly reversed by EA (Figure 2A, *p* < 0.05, compared with the CFA group, *n* = 6). In contrast, TRPV1 expression remained unaltered in the sham EA knockout (KO) group (Figure 2A, *p* < 0.05, compared with the EA group, *n* = 6). Moving along the signaling cascade, we then analyzed the phosphorylated A (pPKA) protein expression level. We found a similar expression trend between TRPV1 and pPKA in the inflamed mouse PFC. Particularly, pPKA was significantly increased in the CFA group (Figure 2B, *p* < 0.05, *n* = 6), and this increase was reversed in the EA group (Figure 2B, *p* < 0.05, *n* = 6) compared with the CFA group. This phenomenon was not observed in the sham EA group (Figure 2B, *p* < 0.05, *n* = 6). A similar tendency was observed for phosphorylated phosphoinositide 3-kinase (pPI3K) and protein kinase Cε (pPKCε) protein concentrations, with the CFA group showing significant increases in both (Figure 2C,D, *p* < 0.05, *n* = 6). Again, potentiation was significantly reduced in the EA group (Figure 2C,D, *p* < 0.05, *n* = 6) but not in the sham EA group (Figure 2C,D, *p* < 0.05, *n* = 6). These results demonstrate that the downstream intracellular mechanisms of the pPKA/pPI3K/pPKCε pathways are associated with inflammatory pain.

**Figure 3:**
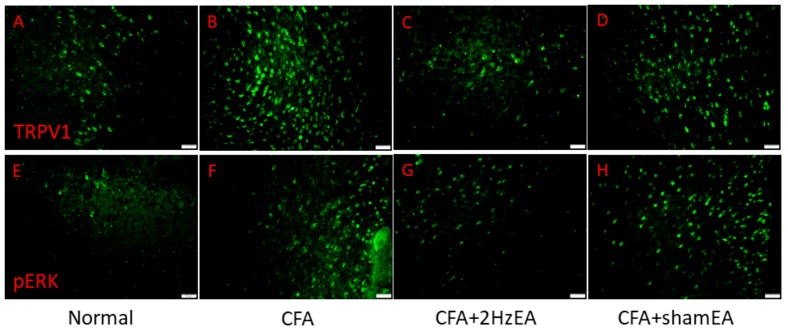
Expressions of TRPV1 and pERK in the mPFC of normal, CFA, CFA + 2 Hz EA, and CFA + sham EA. TRPV1-positive neurons (green) in the mPFC of (**A**) normal, (**B**) CFA, (**C**) CFA + 2 Hz EA, and (**D**) CFA + sham EA mice. pERK-positive neurons (green) in the mPFC of (**E**) normal, (**F**) CFA, (**G**) CFA + 2 Hz EA, and (**H**) CFA + sham EA mice. Scale bar means 50 m.

We then investigated the mitogen activated protein kinase (MAPK) family in the mouse PFC. We found a statistically significant increase in the expression of pERK in the CFA group (*p* < 0.05, *n* = 6; Figure 2E), suggestive of the CFA’s involvement in pERK expression. Notably, this increase was reduced by EA (*p* < 0.05, *n* = 6; Figure 2E) but not by sham EA (*p* < 0.05, *n* = 6; Figure 2E). In addition, pp38 was analyzed as a component of the MAPK signaling pathway. CFA mice presented a significant increase in pp38 protein expression (*p* < 0.05, *n* = 6; Figure 2F), which was reversed by EA (*p* < 0.05, *n* = 6; Figure 2F) but not by sham EA (*p* < 0.05, *n* = 6; Figure 2F). CFA mice also exhibited increased pJNK expression (*p* < 0.05, *n* = 6; Figure 2G), which was reduced by EA (*p* < 0.05, *n* = 6; Figure 2G) but not by sham EA (*p* < 0.05, *n* = 6; Figure 2G). CFA mice also presented significantly increased expression of phosphorylated protein kinase B (pAkt) (*p* < 0.05, *n* = 6; Figure 2H), which was significantly attenuated by EA (*p* < 0.05, *n* = 6; Figure 2H) but not by sham EA (*p* < 0.05, *n* = 6; Figure 2H). The same was found for pmTOR expression (*p* < 0.05, *n* = 6; Figure 2I). The CFA group also presented increased pNFκB expression (*p* < 0.05, *n* = 6; Figure 2J), which was reversed by EA (*p* < 0.05, *n* = 6; Figure 2J) but not by sham EA (*p* < 0.05, *n* = 6; Figure 2J). Both Nav1.7 and Nav1.8 were significantly increased in the PFC of CFA mice (*p* < 0.05, *n* = 6; Figure 2K,L), which was attenuated by EA treatment (*p* < 0.05, *n* = 6; Figure 2K,L, respectively). Similar patterns were observed for immunofluorescence staining. Immunohistochemical staining visualized by green fluorescence indicated that TRPV1 and pERK were expressed in mouse mPFC brain areas and that expression increased after CFA injection. These patterns were reversed by 2 Hz EA manipulation but not by sham manipulation (Figure 3).

**Figure 4:**
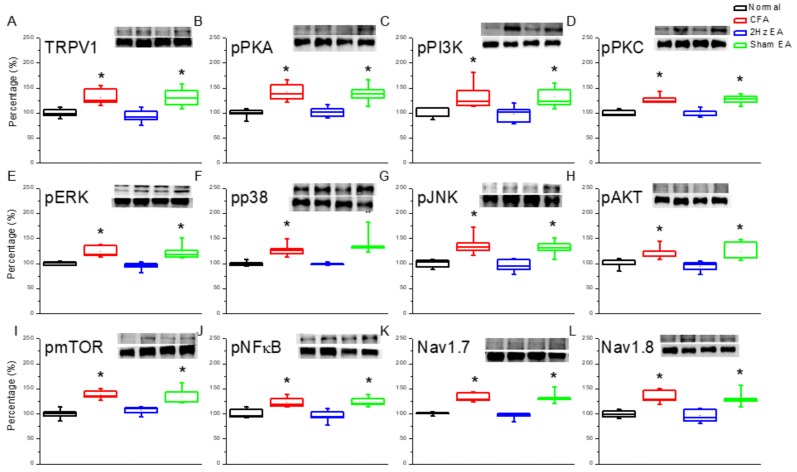
Expression levels of TRPV1-associated signaling pathways in the mice hypothalamus. (**A**) TRPV1, (**B**) pPKA, (**C**) pPI3K, (**D**) pPKCε, (**E**) pERK, (**F**) pp38, (**G**) JNK, (**H**) pAkt, (**I**) pmTOR, (**J**) pNFκB, (**K**) Nav1.7, and (**L**) Nav1.8 expression levels in normal, CFA, CFA + 2 Hz EA, and CFA + sham EA (from left to right). Normal = normal mice; CFA = CFA-induced inflammatory pain; 2 Hz EA = CFA + 2H z EA. Sham EA = CFA + sham EA. *p < 0.05 compared with the normal group. The western blot bands at the top show the target protein. The lower bands are internal controls (β-actin or α-tubulin).

Next, we investigated protein changes in the mouse hypothalamus, which is an important brain region for pain signaling. CFA mice showed increased TRPV1 expression in the hypothalamus (*p* < 0.05, *n =* 6; Figure 4A) compared with the control group (*p* < 0.05, *n =* 6; Figure 4A). The observed TRPV1 increase was dramatically reversed by EA (*p* < 0.05, *n =* 6; Figure 4A) but not by sham EA (*p* < 0.05, *n =* 6; Figure 4A). A similar trend was observed for pPKA protein expression in the CFA group (*p* < 0.05, *n =* 6; Figure 4B) compared with the control group (*n =* 6; Figure 4B). Similarly, this increase was significantly reduced by EA (*p* < 0.05, *n =* 6; Figure 4B) but not by sham EA (*p* < 0.05, *n =* 6, Figure 4B). Similar trends were observed for the pPI3K and the pPKCε (Figure 4C,D) signaling pathways.

**Figure 5:**
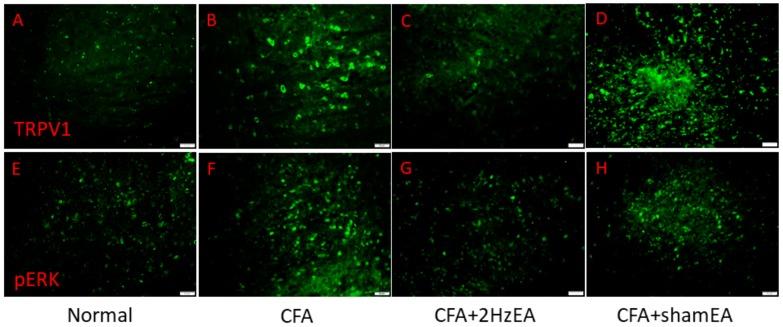
Expressions of TRPV1 and pERK in the hypothalamus of normal, CFA, CFA + 2 Hz EA, and CFA + sham EA. TRPV1-positive neurons (green) in the mPFC of (**A**) normal, (**B**) CFA, (**C**) CFA + 2 Hz EA, and (**D**) CFA + sham EA mice. pERK-positive neurons (green) in the mPFC of (**E**) normal, (**F**) CFA, (**G**) CFA + 2 Hz EA, and (**H**) CFA + sham EA mice. Scale bar means 50 m.

We next investigated the three major MAPK subfamilies. The following results were observed in CFA mice: pERK (*p* < 0.05, *n* = 6, Figure 4E); pp38 (*p* < 0.05, *n* = 6, Figure 4F); and pJNK (*p* < 0.05, *n* = 6, Figure 4G). EA influenced these changes as follows: pERK (*p* < 0.05, *n* = 6, Figure 4E); pp38 (*p* < 0.05, *n* = 6, Figure 4F); and pJNK (*p* < 0.05, *n* = 6, Figure 4G). Such protein content attenuation was not observed in the sham EA group for pERK (*p* < 0.05, *n* = 6, Figure 4E), for pp38 (*p* < 0.05, *n* = 6, Figure 4F), or for pJNK (*p* < 0.05, *n* = 6, Figure 4G). Simultaneously, downstream pPI3K molecules including pAkt and pmTOR were examined in all groups. pAkt showed a similar trend to TRPV1 in that it was increased in the hypothalamus of CFA mice (*p* < 0.05, *n* = 6, Figure 4H) and was attenuated by EA (*p* < 0.05, *n* = 6, Figure 4H) but not by sham EA (*p* < 0.05, *n* = 6, Figure 4H). Similar results were obtained for pmTOR protein expression (*n* = 6; Figure 4I). Furthermore, the expression of pNFκB was significantly increased in CFA mice (*p* < 0.05, *n* = 6; Figure 4J), and this overexpression was attenuated by EA (*p* < 0.05, *n* = 6; Figure 4J) but not by sham EA (*p* < 0.05, *n* = 6; Figure 4J). Accordingly, the expression of Nav1.7 and Nav1.8 in the mouse hypothalamus was also analyzed. For both, expression was potentiated by CFA injection in the mouse hypothalamus (*p* < 0.05, *n* = 6; Figure 4K) and (*p* < 0.05, *n* = 6; Figure 4L, respectively), and this potentiation was dramatically reduced by EA (*p* < 0.05, *n* = 6; Figure 4K–L) but not by sham EA (*p* < 0.05, *n* = 6; Figure 4K–L). Immunofluorescence staining provided consistent results. Immunohistochemical staining visualized by green fluorescence indicated that TRPV1 and pERK were expressed in the hypothalamus and augmented in the CFA group. This phenomenon was attenuated by 2 Hz EA but not by sham EA (Figure 5).

**Figure 6:**
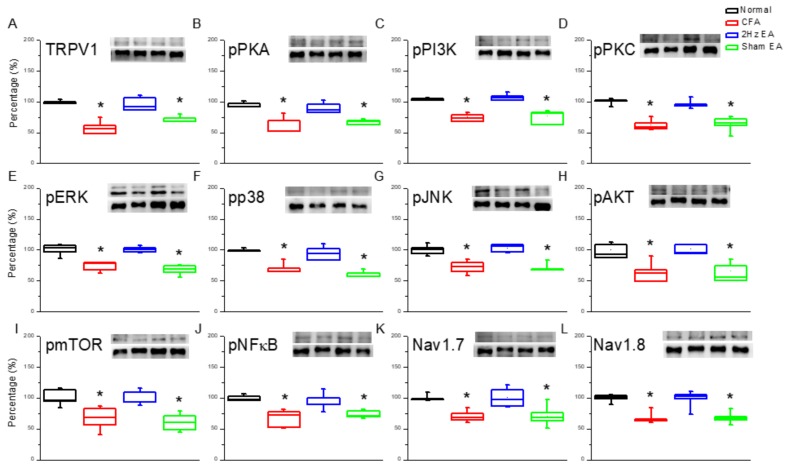
Expression levels of TRPV1-associated signaling pathways in the mice periaqueductal gray (PAG). (**A**) TRPV1, (**B**) pPKA, (**C**) pPI3K, (**D**) pPKCε, (**E**) pERK, (**F**) pp38, (**G**) JNK, (**H**) pAkt, (**I**) pmTOR, (**J**) pNFκB, (**K**) Nav1.7, and (**L**) Nav1.8 expression levels in normal, CFA, CFA + 2 Hz EA, and CFA + sham EA (from left to right). Normal = normal mice; CFA = CFA-induced inflammatory pain; 2 Hz EA = CFA + 2 Hz EA. Sham EA = CFA + sham EA. *p < 0.05 compared with the normal group. The western blot bands at the top show the target protein. The lower bands are internal controls (β-actin or α-tubulin).

To determine the influence of CFA-induced inflammatory pain or EA on pain signaling in the descending pathway, we dissected the PAG for protein analysis. We found that CFA injection significantly reduced the expression of TRPV1 in the PAG (Figure 6A, *p* < 0.05, *n* = 6) compared to controls (Figure 6A, *p* < 0.05, *n* = 6). This attenuation was reliably reversed by 2 Hz EA (Figure 6A, *p* < 0.05, *n* = 6) but not by sham EA (Figure 6A, *p* < 0.05, *n* = 6). We tested the aforementioned molecules in the PAG to clarify their involvement in the descending inhibitory pathway. A similar trend was observed in the pPKA protein expression in CFA mice (*p* < 0.05, *n* = 6; Figure 6B) compared to control mice. This decrease was significantly reversed by EA (*p* < 0.05, *n* = 6; Figure 6B) but not by sham EA (*p* < 0.05, *n* = 6, Figure 6B). Similar trends were observed for the pPI3K and the pPKCε (Figure 6C,D) signaling pathways.

**Figure 7:**
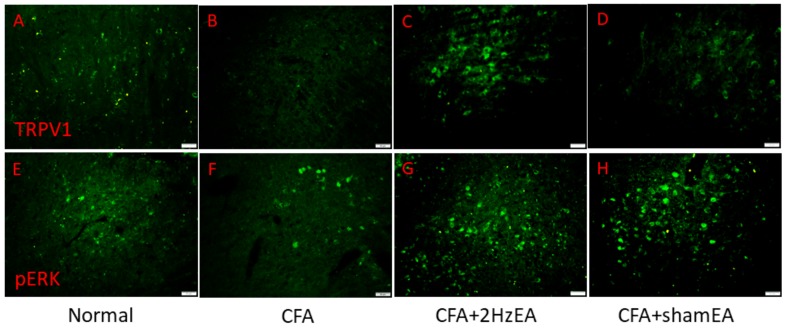
Expressions of TRPV1 and pERK in the PAG of normal, CFA, CFA + 2 Hz EA, and CFA + sham EA. TRPV1-positive neurons (green) in the mPFC of (**A**) normal, (**B**) CFA, (**C**) CFA + 2 Hz EA, and (**D**) CFA + sham EA mice. pERK-positive neurons (green) in the mPFC of (**E**) normal, (**F**) CFA, (**G**) CFA + 2 Hz EA, and (**H**) CFA + sham EA mice. Scale bar means 50 m.

Next, we tested the three major MAPK subfamilies in the PAG. The following results were obtained for CFA mice: pERK (*p* < 0.05, *n* = 6, Figure 6E); pp38 (*p* < 0.05, *n* = 6, Figure 6F); and pJNK (*p* < 0.05, *n* = 6, Figure 6G). EA influenced those changes as follows: pERK (*p* < 0.05, *n* = 6, Figure 6E); pp38 (*p* < 0.05, *n* = 6, Figure 6F); and pJNK (*p* < 0.05, *n* = 6, Figure 6G). Such protein content attenuation was not observed in the sham EA group for pERK (*p* < 0.05, *n* = 6, Figure 6E), for pp38 (*p* < 0.05, *n* = 6, Figure 6F), or for pJNK (*p* < 0.05, *n* = 6, Figure 6G). Simultaneously, downstream pPI3K molecules including pAkt and pmTOR were examined in all groups. pAkt showed a similar trend to TRPV1, since it was decreased in the PAG of CFA mice (*p* < 0.05, *n* = 6, Figure 6H), and that decrease was reversed by EA (*p* < 0.05, *n* = 6, Figure 6H) but not by sham EA (*p* < 0.05, *n* = 6, Figure 6H). Similar results were obtained for pmTOR protein expression (*n* = 6; Figure 6I). Furthermore, the expression of pNFκB was significantly decreased in CFA mice (*p* < 0.05, *n* = 6; Figure 6J), and this overexpression was reversed by EA (p < 0.05, *n* = 6; Figure 6J) but not by sham EA (*p* < 0.05, *n* = 6; Figure 6J). The expression of Nav1.7 and Nav1.8 in the mouse PAG was also analyzed. For both, expression was reduced by CFA injection in the mouse PAG (*p* < 0.05, *n* = 6; Figure 6K) and (*p* < 0.05, *n* = 6; Figure 6L, respectively), and this effect was dramatically reversed by EA (*p* < 0.05, *n* = 6; Figure 6K,L) but not by sham EA (*p* < 0.05, *n* = 6; Figure 6K,L). Similar results were observed for immunofluorescence staining, which indicated that TRPV1 and pERK were expressed in the PAG and reduced in the CFA group. This phenomenon was attenuated by 2 Hz EA but not by sham EA (Figure 7).

## 3. Discussion

**Figure 8:**
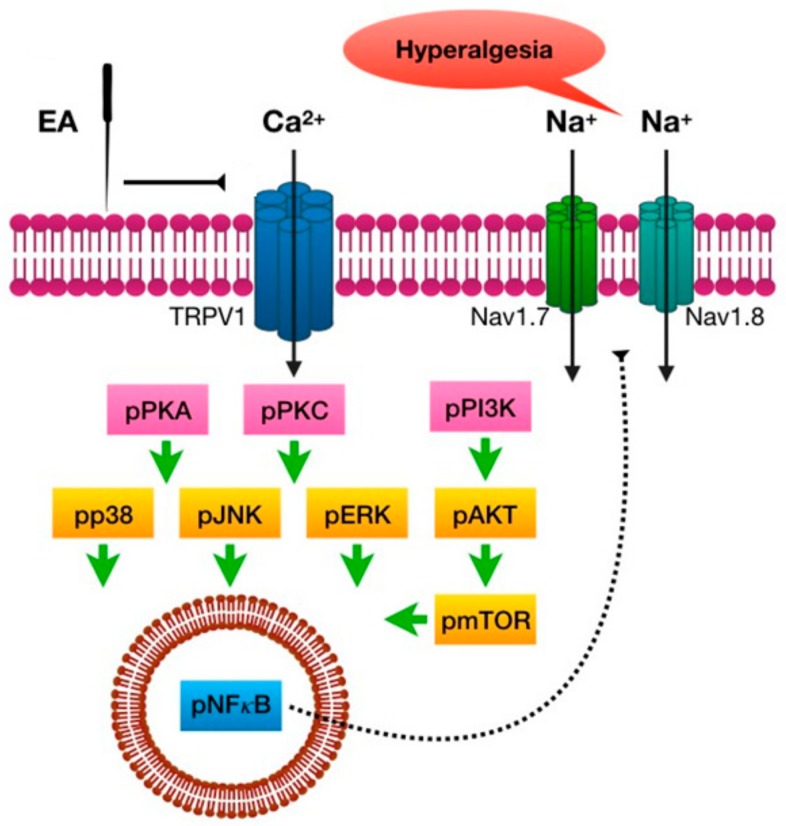
Schematic illustration of distal EA mechanisms of analgesia in CFA-induced inflammatory pain. Summary diagram of how distal EA and TRPV1 are crucial for inflammatory pain and related mechanisms. Our results suggest that distal EA can reduce inflammatory pain through brain mechanisms.

The present study shows, for the first time, that EA at the distal acupoint LI4 significantly reduces inflammation-initiated mechanical and thermal pain. CFA injection induced TRPV1 and related signals in the mouse PFC and hypothalamus, which were reversed by EA treatment but not by sham EA. In addition, TRPV1 and associated molecules were attenuated in the inflamed mice group, which was also abolished by EA but not by sham EA. Taken together, our results provide the first evidence of a TRPV1-mediated distal EA signaling mechanism (Figure 8).

While much is known about the application of local acupuncture in pain treatment, the effects of distal acupuncture, particularly its detailed mechanisms, remain poorly understood [26,27]. Acupuncture-related analgesia is reported to diffuse noxious inhibitory control [28] with the principle that it inhibits C-fibers [29]. Schliessbach et al. suggested that acupuncture significantly increases the pressure pain detection threshold [30]. Yang et al. reported that EA applied at the ipsilateral stomach 36 (ST36) has a better analgesic effect in carrageenan-induced arthritic pain than at the contralateral SP6. Acupuncture’s antinociceptive effect is believed to occur via μ-opioid receptors, since it is reliably blocked by injection of a μ-opioid antagonist [31]. In a clinical trial of carpal tunnel syndrome, Maeda et al. demonstrated that EA at pericardium 7 (PC7) and triple warmer 5 (TW5) exerted similar analgesic effects to EA at distal spleen 6 (SP6) and liver 4 (LV4) compared to a sham control group [27]. Their results showed that local EA produced greater activation of insula and S2, while distal EA produced greater activation in S2 and deactivation of the posterior cingulate cortex. Moreover, distal EA significantly activated pain reduction mediator PFC signals [27].

Our results indicate that both ipsilateral and contralateral EA significantly reduced CFA-induced inflammatory pain. We also showed that the expression of TRPV1 and associated signaling pathways increased after CFA injection and were attenuated by EA. Potentiation of TRPV1 and its associated signaling pathway can be prevented in TRPV1 KO mice, which suggests that TRPV1 KO mice are resistant to inflammatory pain [10]. TRPV1 is reported to excite the central nervous system [32]. ELISA tests have shown that, in normal conditions, TRPV1 is expressed in striatum, hypothalamus, amygdala, hippocampus, substantia nigra, etc [1], and it is known to play roles in inflammatory pain or harmful stimuli [33]. Liao et al. reported that capsaicin injection significantly activated TRPV1 receptors, inducing a painful sensation [34]. TRPV1 activation can trigger downstream molecules such as pPKA, pPI3K, pPKC, pAkt, and pmTOR [35]. Phosphorylated kinases further increase the expression of Nav1.7 and Nav1.8 to propagate painful signals [36]. Xing and Li reported that activation of TRPV1 receptors significantly increases neuronal PAG activity via the potentiation of glutamatergic signals [37]. The activation of TRPV1 can deliver an analgesic descending inhibitory pathway in the PAG and may constitute a novel pain control strategy [38]. Using the enhanced green fluorescent protein (EGFP) transgenic method, Lu et al. reported that Nav1.8 is abundant in the hypothalamus, the somatosensory cortex, and the amygdala and is important for pain sensation [36].

EA is known to activate Aβ and Aδ fibers for pain control [39]. Han suggested that high-frequency EA (e.g., 100 Hz) release dynorphin, while low-frequency EA (e.g., 2 Hz) triggers the release of β-endorphin, encephalin, and endomorphin [40]. Using a CFA inflammatory pain model, Zhang et al. reported that EA at gallbladder 30 (GB30) had a similar analgesic effect at 100 and 10 Hz [41]. Silva et al. reported that 2 Hz EA had a better and longer analgesic effect than 100 Hz EA at the ST36 and the SP9 acupoints and could be blocked by the α-adrenoceptor antagonist [42]. CFA injection increases TRPV1 in the DRG and the SC for pain signaling, but its effect at the brain level remains unclear [6]. Chen et al. reported that acid saline injection significantly induces the activation of the TRPV1 downstream molecule pERK in the paraventricular hypothalamic nucleus [43]. Injection of a TRPV1 agonist into the PAG reliably activates glutamatergic nerve pulses that activate “off” cells for pain relief [34]. 

## 4. Materials and Methods

### 4.1. Animals

C57BL/6 female mice aged 8–12 weeks were used. The animals were purchased from BioLASCO Co. Ltd., Taipei, Taiwan and were randomly assigned to one of four groups (*n* = 8 per group): (1) normal, (2) CFA, (3) CFA + 2 Hz EA, and (4) CFA + sham EA. A sample size of eight animals per group was calculated as the number required for an alpha of 0.05 and a power of 80%. After arrival, mice were housed in a 12/12 h light/dark cycle room with water and food available ad libitum. All experimental and husbandry procedures were approved by the Institute of Animal Care and Use Committee of China Medical University (No. 2018-110, 21 December 2017) and conducted according to the Guide for the use of Laboratory Animals provided by the National Research Council and the ethical guidelines of the International Association for the Study of Pain. The number of animals used and their suffering were minimized.

### 4.2. Inflammatory Pain Model

All experiments were performed in our laboratory during daylight hours. The C57B/L6 mice were randomly assigned to one of four groups and put into a fixation machine under anesthesia with 1% isoflurane for inflammatory pain induction and EA treatments. Intraplantar inflammation was induced by injecting mice in the plantar surface of the hind paw with either 20 μL saline (pH 7.4, buffered with 20 mM HEPES) or 0.5 mg/mL CFA (0.5 mg/mL heat-killed *M. tuberculosis* (Sigma, St. Louis, MO, USA) suspended in oil:saline 1:1 emulsion) using a 27-gauge needle. The procedures were performed between 09:00 and 12:00, after which the animals were returned to their home cages.

### 4.3. Electroacupuncture Treatment

Acupuncture needles (0.5 inch, 32 G, Yu Kuang, Bao Feng Tang Gole Medical, Taipei, Taiwan) were inserted into the LI4 acupoint’s muscle layer at a 1–2 mm depth under 1% isoflurane anesthesia. For the sham group, the needle was inserted into the LI4 acupoint without any rotation or twisting. The LI4 acupoint is located on the dorsum of the hand, radial to the midpoint of the forepaw’s second metacarpal bone. To ensure an insertion depth of 1–2 mm, a piece of tape was stuck to the needle, leaving space only enough for manipulation and a needle tip of 2 mm. EA treatment was applied by delivering electrical stimulation of 2 Hz using a Trio 300 electrical stimulator (Grand Medical Instrument Co. Ltd., Japan). Electrical pulses were delivered at 100 μs square pulses of 1 mA for 15 min at 2 Hz.

### 4.4. Animal Behavior

Mechanical and thermal nociception and sensitivity were tested thrice throughout the experiment. All mice were transported to the behavior analysis room and allowed to acclimate for 1 h prior to the behavior tests. All experiments were performed at room temperature (24 ± 2 °C), and the stimuli were only applied when the animals were calm and not sleeping or grooming. First, the von Frey assessment was conducted. Mechanical sensitivity was measured by testing the strength of response to stimulation using three applications of electronic, calibrated von Frey filament on the mice’s hind paw (IITC Life Science Inc., CA, USA). Anesthetized subjects were placed on a metal mesh (75 × 25 × 45 cm) covered with a plexiglass cage (10 × 6 × 11 cm) and acclimated for 1 h. Subjects were then mechanically stimulated by the tip of the filament at the plantar region of the right or the left hind paw. Filament gram counts were recorded when the stimulation caused the subject to withdraw its hind paw. A cut-off pressure of 20 g was set to avoid tissue damage. Thermal pain was measured by testing the response time to thermal stimulation with six applications using Hargreaves’ test IITC analgesiometer (IITC Life Sciences, SERIES8, Model 390G, CA, USA). The thermal stimulator was positioned under the glass sheet, and the focus of the projection bulb was aimed exactly at the middle of the plantar surface of the right or the left hind paw. A mirror attached to the stimulator allowed the plantar surface to be visualized. A cut-off time of 20 s was set to prevent tissue damage. In the thermal paw withdrawal test, the nociception threshold was assessed using the latency of paw withdrawal upon stimulus and was recorded when the constant applied heat stimulation caused the subject to withdraw its hind paw. 

### 4.5. Tissue Sampling and Western Blot Analysis

The PFC, the hypothalamus, and the PAG were immediately excised to extract proteins. Total proteins were prepared by homogenizing the tissues in lysis buffer containing 50 mM Tris-HCl (pH 7.4), 250 mM NaCl, 1% NP-40, 5 mM EDTA, 50 mM NaF, 1 mM Na_3_VO_4_, 0.02% NaNO_3_, and 1 × protease inhibitor cocktail (AMRESCO). The extracted proteins (30 μg per sample according to the bicinchoninic acid (BCA) protein assay) were subjected to 8% SDS-tris glycine gel electrophoresis and transferred onto a polyvinylidene difluoride (PVDF) membrane. The membrane was blocked with 5% non-fat milk in TBS-T buffer (10 mM tris-buffered saline, pH 7.5, 100 mM NaCl, 0.1% Tween 20), incubated with the first antibody in TBS-T and 1% bovine serum albumin, and incubated for 1 h at room temperature. A peroxidase-conjugated anti-rabbit antibody (1:5000) was used as the secondary antibody. The bands were visualized using an enhanced chemiluminescent substrate kit (PIERCE) with LAS-3000 Fujifilm (Fuji Photo Film Co. Ltd., Japan). Specific bands’ image intensities were quantified with NIH ImageJ 1.52A (Bethesda, Wisconsin, MD, USA), as appropriate. Protein ratios were obtained by dividing the target protein intensities by the intensity of α-tubulin in the same sample. Calculated ratios were then adjusted by dividing the ratios from the same comparison group relative to the control.

### 4.6. Immunofluorescence

Mice were anesthetized with 1% isoflurane and intracardially perfused, first with normal saline followed by 4% paraformaldehyde. Mice brains were then placed in 30% sucrose and embedded in tissue optimum cutting temperature (OCT)-freeze medium at –20°C on the following day. Frozen sections were cut (20 μm) and placed on amino propyltriethoxy silane (APS)-coated glass microslides. Subsequently, the sections were post-fixed in 4% paraformaldehyde for 3 min and incubated in blocking solution containing 3% bovine serum albumin (BSA, Merck, USA), 0.1% Triton X-100, and 0.02% NaN3 in phosphate buffered saline (PBS) for 2 h at room temperature. After blocking, brain sections were incubated with primary antibodies in a blocking solution at 4 °C overnight. The following primary antibodies were used: anti-TRPV1 (1:500, Alomone, Israel) and anti-pERK (1:500, Alomone, Israel) from Alomone. The secondary antibody was a goat anti-rabbit (1:500) antibody (MolecularProbes, Carlsbad, CA, USA). Slides were mounted with cover slips and visualized using a fluorescence microscope (CKX41 with an Olympus U-RFLT50 Power Supply Unit, Olympus, Tokyo, Japan).

### 4.7. Statistical Analysis

All data were expressed as the mean ± standard error. Significant differences between the normal, the CFA, the CFA + 2 Hz EA, and the CFA + sham EA groups were tested using ANOVA followed by a post hoc Tukey’s test. A level of *p* < 0.05 was considered significantly different. The Shapiro–Wilk and the Levene tests were used to investigate data distribution and variance homogeneity, respectively.
